# AXL Controls Directed Migration of Mesenchymal Triple-Negative Breast Cancer Cells

**DOI:** 10.3390/cells9010247

**Published:** 2020-01-19

**Authors:** Olivier Zajac, Renaud Leclere, André Nicolas, Didier Meseure, Caterina Marchiò, Anne Vincent-Salomon, Sergio Roman-Roman, Marie Schoumacher, Thierry Dubois

**Affiliations:** 1 Breast Cancer Biology Group, Translational Research Department, Institut Curie, PSL Research University, 75005 Paris, France; olivier.zajac@curie.fr; 2 Department of Pathology, Platform of Investigative Pathology, Institut Curie, PSL Research University, 75005 Paris, France; renaud.leclere@curie.fr (R.L.); andre.nicolas@curie.fr (A.N.); didier.meseure@curie.fr (D.M.); 3 Department of Medical Sciences, University of Turin, Via Verdi 8, 10124 Torino TO, Italy; caterina.marchio@unito.it; 4 Department of Pathology, Institut Curie, PSL Research University, 75005 Paris, France; anne.salomon@curie.fr; 5 Translational Research Department, Institut Curie, PSL Research University, 75005 Paris, France; sergio.roman-roman@curie.fr; 6 Center for Therapeutic Innovation Oncology, Institut de Recherches Internationales SERVIER, 92284 Suresnes, France; marie.schoumacher@servier.com

**Keywords:** AXL, migration, polarity, triple-negative breast cancer, directionality, R428

## Abstract

Triple-negative breast cancer (TNBC) is an aggressive form of breast cancer with high risk of relapse and metastasis. TNBC is a heterogeneous disease comprising different molecular subtypes including those with mesenchymal features. The tyrosine kinase AXL is expressed in mesenchymal cells and plays a role in drug resistance, migration and metastasis. We confirm that AXL is more expressed in mesenchymal TNBC cells compared to luminal breast cancer cells, and that its invalidation impairs cell migration while having no or little effect on cell viability. Here, we found that AXL controls directed migration. We observed that AXL displays a polarized localization at the Golgi apparatus and the leading edge of migratory mesenchymal TNBC cells. AXL co-localizes with F-actin at the front of the cells. In migratory polarized cells, the specific AXL inhibitor R428 displaces AXL and F-actin from the leading edge to a lateral area localized between the front and the rear of the cells where both are enriched in protrusions. In addition, R428 treatment disrupts the polarized localization of the Golgi apparatus towards the leading edge in migratory cells. Immunohistochemical analysis of aggressive chemo-resistant TNBC samples obtained before treatment reveals inter- and intra-tumor heterogeneity of the percentage of AXL expressing tumor cells, and a preference of these cells to be in contact with the stroma. Taken together, our study demonstrates that AXL controls directed cell migration most likely by regulating cell polarity.

## 1. Introduction

Triple negative breast cancer (TNBC) accounts for about 15% of breast cancers and is defined by the lack of expression of estrogen receptor (ER) and progesterone receptor (PR), and the absence of Her2 overexpression [1,2]. A pivotal challenge in the treatment of TNBC patients is their inter- and intra-tumor heterogeneity [3,4,5]. TNBC encompasses a heterogeneous group including tumors with a mesenchymal phenotype [3,6,7]. TNBC is associated with poor prognosis, as a consequence of a high rate of relapse and metastases following chemotherapy treatment [8,9,10].

The ability of some epithelial cells to acquire a mesenchymal phenotype is important during development and wound healing [11,12,13]. This cellular plasticity, called epithelial-to-mesenchymal transition (EMT), allowing the cells to gain new abilities to migrate and cross an extracellular matrix, is crucial for the metastatic process [14,15,16,17]. In addition, cells undergoing EMT are less proliferative and more prone to resist to chemo- and targeted-therapies [18,19,20,21].

AXL, first identified in chronic myelogenous leukemia patients [22], belongs along with TYRO3 and MERTK to the TAM family of receptor tyrosine kinases (RTK) [23]. Its name derives from the Greek term “anexelekto”, which means uncontrolled. AXL can be activated by its ligand, the growth arrest specific protein 6 (GAS6) [24,25] or through its interaction with other transmembrane receptors such as human epidermal growth factor receptor 1 (EGFR) in TNBC [26]. AXL activation leads to its autophosphorylation, switching on downstream signaling cascades such as PI3K/AKT, MAPK, JAK/STAT, SRC and RHO pathways [27,28]. The involvement of AXL in EMT has been largely documented [29,30,31,32,33], and it is likely through this property that AXL plays a role in migration, invasion, drug resistance and metastases [28,29,34,35,36,37,38,39,40,41,42,43,44,45]. For these reasons, a large number of AXL inhibitors have been developed [46]. So far, bemcentinib (R428) is the only inhibitor specific to AXL [47,48].

AXL has been proposed as a potential therapeutic target for breast cancers [29,30,47,49] and its inhibition sensitizes cells to chemo- or targeted-therapies [36,38]. Among breast cancer cell lines, AXL tends to be more expressed in TNBC, and more specifically in mesenchymal TNBC cells [38,50].

To our knowledge, there is no study describing the intracellular localization of AXL. By using immunofluorescence, we have examined the intracellular localization of AXL and shown that AXL displays a polarized localization in mesenchymal TNBC cells. AXL is present at the leading edge and at the Golgi apparatus of migrating cells. AXL inhibition perturbs its polarized localization and impairs directional migration. Altogether, these results provide novel insights about the implication of AXL during the migration of mesenchymal cells. 

## 2. Methods

### 2.1. Cell Lines, Cell Authentication and Cell Culture

Cell lines were purchased from the American Type Culture Collection (ATCC, LGC Promochem, Molsheim, France) and authenticated (data not shown) by short tandem repeat profiling in 2018 using the PowerPlex^®^ 16 System (Promega, Charbonnieres les bains, France; DC6531) [51]. Cells lines were cultured as previously described [52,53,54]. Briefly, Hs578t and MDA-MB-157 cells were cultured in DMEM (Thermo Fisher Scientific, Courtaboeuf, France; 31966047) supplemented with 10% fetal bovine serum (FBS), 100 U/mL penicillin and 100 µg/mL streptomycin (P/S; Thermo Fisher Scientific, Courtaboeuf, France; 10378016). BT-474 and MDA-MB-436 cells were maintained in RPMI-1640 (Thermo Fisher Scientific, Courtaboeuf, France; 61870044) supplemented with 10% and P/S. MDA-MB-231 cells were cultured in DMEM-F12 (Thermo Fisher Scientific, Courtaboeuf, France; 31331093) supplemented with 10% FBS and P/S. MCF7 cells were cultured in MEM (Sigma-Aldrich, Saint-Quentin Fallavier, France; M2279) containing 10% FBS, 1.5 g/L sodium bicarbonate, 0.1 mM non-essential amino acids (Thermo Fisher Scientific, Courtaboeuf, France; 11140050), 1 mM sodium pyruvate and P/S. We maintained all cell lines at 37 °C in a humidified atmosphere with 5% CO_2_.

### 2.2. Protein Extraction

Cell lysates were obtained using a Laemmli buffer containing 50 mM Tris pH 6.8, 2% sodium dodecyl sulfate, 5% glycerol, 2 mM 1,4-dithio-dl-threitol (DTT), 2.5 mM ethylene diaminetetraacetic acid, 2.5 mM ethylene glycol tetraacetic acid, 2 mM sodium orthovanadate, 10 mM sodium fluoride, a cocktail of protease (Roche, Meylan, France; 04693159001) and phosphatase (Thermo Fisher Scientific, Courtaboeuf, France; 1861277) inhibitors, and boiled at 100 °C for 10 min. Protein concentration was calculated using the reducing agent-compatible version of the BCA Protein Assay kit (Thermo Fisher Scientific, Courtaboeuf, France; 23252).

### 2.3. Immunoblot

Proteins were fractionated by SDS-PAGE under reducing conditions (4–15% TGX gels, BioRad, Marnes la Coquette, France; 456-8053) and blotted onto nitrocellulose membranes (BioRad, Marnes la Coquette, France; 1704159). The membranes were blocked with 5% BSA in TBS containing 0.1% Tween20 (TBS-T) and hybridized with the primary antibody of interest overnight at 4 °C. Then, the membranes were washed in TBS-T and hybridized with the secondary antibody for 1 h at room temperature. Antibodies were diluted in TBS-T containing 5% BSA. After washes, immune complexes were revealed by chemiluminescence (Thermo Fisher Scientific, Courtaboeuf, France; 34580), imaged using the ChemiDoc™ Imaging Systems (BioRad, Marnes la Coquette, France) and analyzed with the Image Lab Software (BioRad, Marnes la Coquette, France).

### 2.4. Small Interfering RNAs (siRNAs) and Transfection

Cells were seeded into six well-plates (TPP, Trasadingen, Switzerland; 92106) and next day, transfection was performed with 20 nM small interfering RNAs (siRNA) using INTERFERin reagent (Polyplus, Ozyme, Saint Quentin en Yveline, France; 409-50) in Opti-MEM medium (Thermo Fisher Scientific, Courtaboeuf, France; 31985070), according to the manufacturers’ instructions. For immunofluorescence, cells were plated one day post transfection on a coverslip for 2 days before fixation. The siRNAs used were as follows: Allstars negative control (Qiagen, Courtaboeuf, France; SI03650318), AXL#9 (Qiagen, Courtaboeuf, France; SI00605304) and AXL#10 (Qiagen, Courtaboeuf, France; SI00605311).

### 2.5. Random Migration

Hs578t and MDAMB231 cells were plated in a 24 well plate (TPP, Trasadingen, Switzerland; 92424). Imaging was started one hour following treatment with DMSO or R428 (Sellekchem, Euromedex, Mundolsheim, France; S2841), or 3 days after siRNA transfection. One image every 20 min were acquired using transmitted light for 6 h using IncuCyte^®^ ZOOM Live-Cell Analysis System. Individual cells were manually tracked with the ImageJ plugin Manual Tracking. Directionality was calculated with the ImageJ plugin Chemotaxis and migration tool.

### 2.6. Cell Survival Assays

Cell survival was assessed using CellTiter-Glo^®^ Luminescent Cell Viability Assay (Promega, Charbonnieres les bains, France; G7570) according to the manufacturer’s instructions. Briefly, a volume of CellTiter-Glo reagent medium was added (*v*/*v*). The plates were mixed and incubated 10 min at room temperature. Then luminescence was monitored using Tecan Infinite 200.

### 2.7. Immunoprecipitation (IP)

Cells were cultured with serum prior to treatment with R428 or serum-starved prior to stimulation with Gas6. Cells were lysed in a cold lysis buffer (100 mM NaCl, 50 mM Tris-HCl, pH 7.4, 1 mM EDTA, 1 mM EGTA, 1% NP40, 10% glycerol, DTT 1M and protease and phosphatase inhibitors) under rotation for one hour at 4 °C. The cellular debris were removed by centrifugation at 13,000 rpm for 10 min at 4 °C. 1 µg of AXL-goat antibody or 1 µg of Goat IgG (negative control) were incubated with 1 mg of protein lysate overnight at 4 °C with rotation. Protein G-coupled beads (Thermo Fisher Scientific, Courtaboeuf, France; 20398) were then added for 1 h incubation under rotation at 4 °C. Beads were washed four times with cold lysis buffer before extraction with Laemmli buffer for 10 min at 100 °C. Immunoprecipitated proteins were separated by SDS-PAGE as indicated in the immunoblot section (See above).

### 2.8. Immunofluorescence (IF)

Samples were washed in PBS and fixed in 4% PFA for 15 min. Permeabilization was performed in PBS supplemented with 0.2% BSA and 0.05% saponin for 10 min. Primary antibodies were incubated overnight at 4 °C in a humidified chamber. After 3 washes of 5 min in TBS Tween 0.05%, secondary antibodies and DAPI (1 μg/mL) were incubated one hour at room temperature. Slides were mounted using VectaMount (Vector Laboratories, Eurobio Scientific, Les Ulis, France; H5501). Acquisitions were done using an epifluorescence microscope Leica DM6000B and analyzed using ImageJ.

### 2.9. Wound Assays

Cells were grown on glass coverslips (Marienfeld, VWR, Fontenai-sous-Bois, France; 01115220) in culture-insert 2 well (Ibidi, CliniSciences, Nanterre, France; 80209). Once confluency was reached, cells were treated with DMSO or R428 for one hour before removing the insert to allow the cells to migrate, and starved or stimulated with Gas6 (R&D systems, Lille, France; 885GSB) at 400 ng/mL. To analyze Golgi orientation, cells were fixed after 4 h of migration and immunostained with anti-GM130 antibody and DAPI to visualize the Golgi and the nucleus. Cells were scored as polarized when the Golgi was found facing towards the wound.

### 2.10. AXL Localization Analysis

Cells were plated on coverslip for 3 days and then treated with DMSO (CTRL) or R428 (0.5, 1 or 2 µM), Ly294002 (10 µM, Sellekchem, Euromedex, Mundolsheim, France; S1105), NSC-23766 (50 μM, Tocris, Bio-Techne SAS, Noyal Châtillon sur Seiche, France; 2161), PP2 (10 µM, Sellekchem, Euromedex, Mundolsheim, France; S7008), Dasatinib (10 µM, Sellekchem, Euromedex, Mundolsheim, France; S1021) and Brefeldin A (10 µg/mL, Sellekchem, Euromedex, Mundolsheim, France; S7046). Treatments were performed during 2 h, or 30 min for the Brefeldin A and 30 min, 1-, 2- and 4-h for the R428.

### 2.11. Patient Samples and Multiplexed Immunohistochemistry (IHC)

Core biopsy samples from a series of TNBC patients who underwent neoadjuvant treatment at Institut Curie were retrieved from the archives of the Pathology Department (Table S1). Cases were selected based on the presence of residual disease following neoadjuvant treatment, thus representing chemo-resistant TNBC (Table S1). Experiments were performed in accordance with Bioethics Law No. 2004-800 and the Ethics Charter of the French National Cancer Institute (INCa), with the approval of the ethics committee of our institution. Informed consent was not required. The women were informed of the use of their tissues for research and did not oppose this research.

Three-micron thick serial sections were cut for subsequent immunophenotypical analyses that were performed on a Leica Bond RX Automated IHC stainer. Slides were first dewaxed using Bond Dewax solution (Leica Biosystems, Newcastle, UK; AR9222). Next, we used HIER1 (pH = 6) solution (Leica Biosystems, Newcastle, UK; AR9961) to perform heat induced epitope retrieval. Endogenous peroxidases were blocked using REAL Peroxidase-Blocking solution (Dako France, Agilent, Les Ulis, France; S2023) for 10 min followed by incubation with Protein Block Serum-Free solution (Dako France, Agilent, Les Ulis, France; X0909) for 10 min. Multiplex was done using Opal 7-Color Automation IHC Kit (PerkinElmer, Courtaboeuf, France; NEL821001KT). Primary antibody against AXL was used at 1/300 and was incubated for 1 h at room temperature followed by OpalTM Polymer HRP and Opal620 Reagent according to manufacturer instructions. Then, we used HIER1 (pH = 6) solution to remove antibodies construction (15 min, 95 °C) and we performed the second step of multiplex with pan-keratin AE1/AE3 antibody (1/500, 45 min, 24 °C) revealed by Opal650 Reagent. Images were acquired on a Vectra3 from PerkinElmer. We used InForm software (PerkinElmer, Courtaboeuf, France) to distinguish stroma from tumor areas based on keratin positivity signal and AXL intensity was measured in tumor cells. Analysis of cell in contact with the stroma was done using ImageJ.

### 2.12. Antibodies and Staining

AXL (IHC-IF, Cell Signaling, Ozyme, Saint-cyr-l’école, France; 8661), AXL (IF-IP, R&D systems, Lille, France; AF154), AXL (WB, Cell Signaling, Ozyme, Saint-cyr-l’école, France; 4939), Vimentin (BD Biosciences, Le pont de claix, France; 550513), E-cadherin (BD Biosciences, Le pont de claix, France; 610181), GAPDH (Cell Signaling, Ozyme, Saint-cyr-l’école, France; 2118), Phalloidin (Thermo Fisher Scientific, Courtaboeuf, France; R37112), GM130 (R&D systems, Lille, France; NBP1-89757), LAMP1 (Cell Signaling, Ozyme, Saint-cyr-l’école, France; 9091T), pan-keratin AE1/AE3 (Dako France, Agilent, Les Ulis, France; M3515), (Cell Signaling, Ozyme, Saint-cyr-l’école, France; 9491) and anti-goat IgG (Sigma-Aldrich, Saint-Quentin Fallavier, France; SAB3700257).

### 2.13. Statistics

Significance was tested with unpaired two-tailed Student’s *t*-test using GraphPad Prism (GraphPad, San Diego, CA, USA). The method used, *p* values and n numbers are indicated in the figure legends. *p* values of significance are represented as *** *p* < 0.001, ** *p* < 0.01 and * *p* < 0.05. The exact value is indicated when possible. All graphs represent mean ± s.d.

## 3. Results

### 3.1. AXL Controls Directed Migration in Mesenchymal TNBC Cell Lines

**Figure 1:**
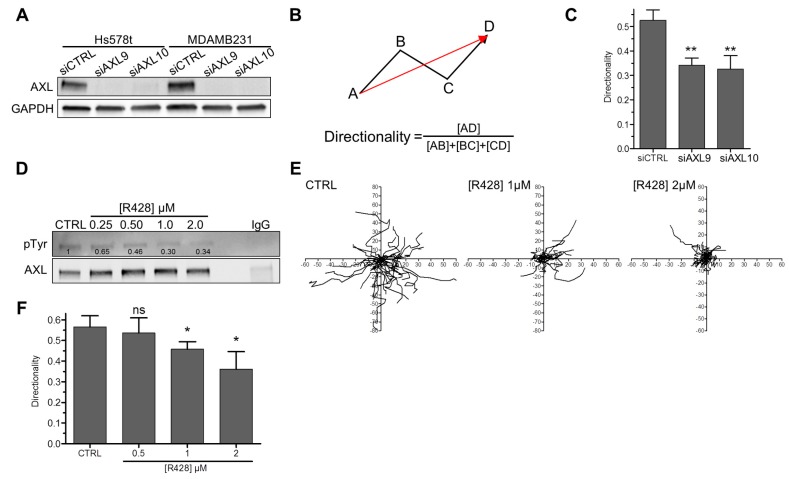
AXL controls directed cell migration. (**A**) AXL protein expression by western blotting in Hs578t and MDA-MB-231 cells three days following transfection with CTRL, AXL9 or AXL10 small interfering RNAs (siRNA). GAPDH was used as a loading control. (**B**) Schematic representation of the method used to measure cell directionality. (**C**) Evaluation of the directionality of Hs578t cells three days after transfection with CTRL, AXL9 or AXL10 siRNA obtained from 110, 100 and 113 cells in three independent experiments, respectively. (** *p* = 0.003; 0.007). (**D**) Hs578t cells were cultured with serum and treated with DMSO (CTRL) or various concentrations (0.25, 0.5, 1 or 2 µM) of R428 for 6 h. Basal phosphorylated active AXL was then detected by western blotting using an anti-phosphotyrosine antibody after AXL immunoprecipitation. As a negative control, IgG instead of AXL antibodies were used with cells treated with DMSO. (**E**) Representative migration trajectories of Hs578t cells treated with DMSO (CTRL) or various concentrations (1 or 2 µM) of R428 for 6 h. (**F**) Directionality of Hs578t cells treated with DMSO (CTRL) or various concentrations (0.5, 1 or 2 µM) of R428 obtained from 108, 96, 80 and 75 cells in three independent experiments, respectively. (ns > 0.05, * *p* = 0.012; 0.024). All graphs represent means and small bars indicate standard deviation.

We assessed AXL expression by western-blot in five mesenchymal TNBC cell lines (BT549, MDA-MB-436, MDA-MB-231, MDA-MB-157 and Hs578t) as well as in one ER-positive/HER2-positive (BT474) and one ER-positive/HER2-negative (MCF7) epithelial luminal cell lines. As expected, the mesenchymal cell lines express Vimentin (a mesenchymal marker) and no/low E-cadherin (an epithelial marker), in contrast to the luminal epithelial cells (Figure S1A in Supplementary Materials). We found that AXL is more expressed in mesenchymal TNBC cells compared to the two luminal cell lines (Figure S1A) confirming previous studies [38]. MDA-MB-231 and Hs578t cells, which display the highest levels of AXL, were chosen for further analyses (Figure S1A). By using two distinct siRNA targeting AXL (Figure 1A), we found that AXL depletion in MDA-MB-231 and Hs578t cell lines impairs cell motility (Figure S1B) but not cell viability/proliferation (Figure S1C), in agreement with published data [33,47,49,55,56,57,58]. We next investigated whether AXL invalidation affects directed (or oriented) cell migration (Figure 1B). The depletion of AXL in Hs578t (Figure 1C) and MDA-MB-231 (Figure S1D) cells decreased the directionality of cell migration. We next investigated whether the kinase activity of AXL was required for cell migration directionality. First, we confirmed that specific inhibition of AXL, using the small molecule R428, impairs basal AXL tyrosine phosphorylation (Figure 1D and Figure S1E) and cell motility (Figure S1F) in a dose dependent manner in our cellular models. Similarly to AXL depletion (Figure 1C and Figure S1D), AXL inhibition disturbed the directionality of cell migration of Hs578t (Figure 1E,F) and MDA-MB-231 cells (Figure S1G). 

Taken together, our results confirmed that AXL invalidation impairs cell motility. Most importantly, we found that AXL controlled directed migration.

### 3.2. Polarized Localization of AXL at the Leading Edge and the Golgi Apparatus in Migrating Cells

**Figure 2:**
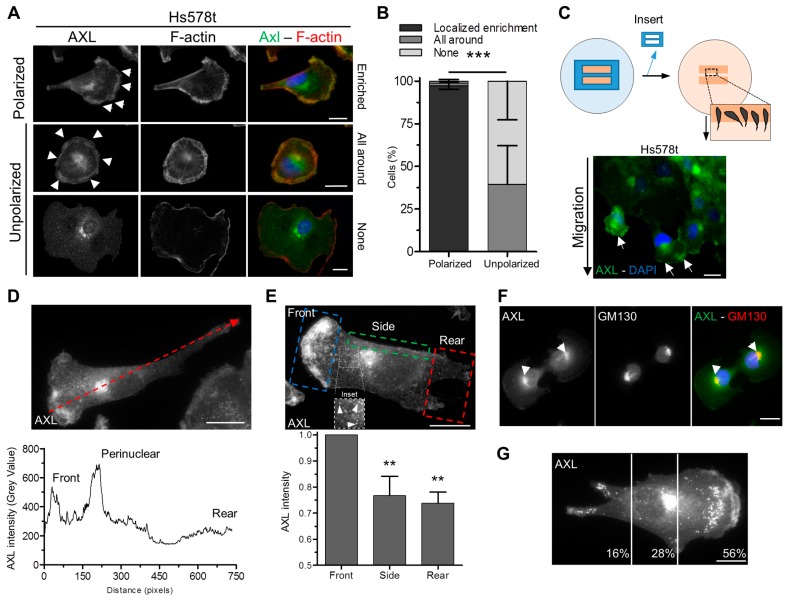
Polarized localization of AXL in migrating cells. (**A**) Staining of AXL (green) and F-actin (red) in polarized or unpolarized Hs578t cells. “All around” defines cells with AXL enriched all along the plasma membrane; “None” defines cells without AXL at the plasma membrane. Arrowheads point to AXL enriched staining at the plasma membrane. (**B**) Frequency of polarized (left column) or unpolarized (right column) Hs578t cells (%) displaying or not an enrichment of AXL at the plasma membrane (localized enrichment, all around) from the analysis of 211 cells in three independent experiments. (*** *p* < 0.001). (**C**) Upper panel: schematic representation of a wound healing experiment. Lower panel: AXL staining (green) in Hs578t cells six hours after wound healing. Arrows point to AXL enrichment at the front of migrating cells. (**D**) Linescan of AXL intensity (grey value) of Hs578t cells (following the dashed red arrow on the top image). (**E**) AXL intensity at the lateral area and at the rear of Hs578t cells relative to the front obtained from 42 cells in four independent experiments. (** *p* = 0.008; 0.0011). Arrowheads in inset point to AXL-stained structures. (**F**) AXL (green) and GM130 (red) staining of Hs578t cells. Arrowheads point to AXL staining at the perinuclear area. (**G**) Percentage of AXL-rich vesicles in polarized cells depending on their localization obtained from 20 cells in two independent experiments. Scale bars, 20 µm. All graphs represent means and small bars indicate standard deviation.

In order to better understand how AXL regulates cell migration and its directionality, we then analyzed its localization by immunofluorescence in mesenchymal TNBC cell lines. First, we validated two different AXL antibodies (AF154 ab and C89E7 ab) for immunofluorescence staining in Hs578t cells depleted or not for AXL (Figure S2A). Both antibodies gave similar results with a perinuclear and a plasma membrane enriched localization of AXL, which decreased in AXL depleted Hs578t cells (Figure S2A). Further experiments were then performed using the AXL AF154 ab, which gave stronger signals. We observed that AXL localization at the plasma membrane depended on the polarized state of Hs578t cells (Figure 2A). Indeed, AXL was undetectable or present all around the plasma membrane in non-polarized cells whereas it displayed enrichment at a specific region resembling to a leading edge when the cells were polarized (Figure 2A,B). To confirm the presence of AXL at the front of migratory cells, a wound healing experiment was performed revealing that AXL is indeed enriched at the leading edge of migrating Hs578t (Figure 2C–E) and MDA-MB-231 (Figure S2B) cells.

To better characterize the AXL perinuclear localization (Figure S2A), we performed a co-staining experiment using antibodies against AXL and GM130, a cis-Golgi marker, and we demonstrated that AXL was enriched at the Golgi apparatus (Figure 2F). The treatment of cells with Brefeldin A, which causes the disruption of the Golgi apparatus, led to the loss of AXL at the perinuclear region (Figure S2C). Moreover, we noticed an enrichment of AXL in structures between the cell front and the Golgi apparatus (Figure 2E), which were positives for Lamp1 (Figure S2D), suggesting that AXL was present in vesicles between these two compartments (Figure 2G). 

Taken together, our results highlighted a polarized localization of AXL at the leading edge and at the Golgi apparatus in migratory cells. 

### 3.3. The Inhibition of AXL Disrupts Its Localization at the Leading Edge of Migrating Cells

**Figure 3:**
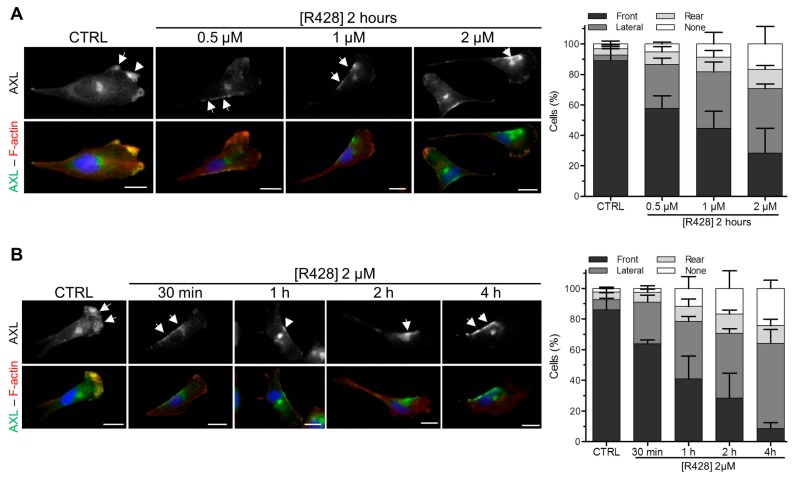
The inhibition of AXL disrupts its localization at the front of migratory cells. (**A**) Hs578t cells were treated with DMSO (CTRL) or various concentrations (0.5, 1 or 2 µM) of R428 for 2 h prior AXL (green) and F-actin (red) staining (left panel). Quantification of the cells (%) presenting a localization of AXL at the front, at the lateral area or at the rear in DMSO- or R428-treated cells (right panel) obtained from 183, 208, 176 and 218 cells in three independent experiments, respectively. (**B**) Hs578t cells were treated with DMSO (CTRL) or 2 µM of R428 during 30 min, 1 h, 2 h or 4 h prior AXL (green) and F-actin (red) staining (left panel). Quantification of the cells (%) presenting a localization of AXL at the front, at the lateral area or at the rear in DMSO- or R428-treated cells for 30 min, 1 h, 2 h or 4 h (right panel) obtained from 216, 181, 190, 218 and 217 cells in three independent experiments, respectively. Arrows point to AXL enriched localization. Scale bars, 20 µm. All graphs represent means and small bars indicate standard deviation.

Next, we explored the consequences of AXL inhibition on its polarized localization in migrating cells. R428 treatment induces the loss of AXL staining at the front of migrating cells at different doses (Figure 3A and Figure S3A) and in a time (Figure 3B and Figure S3B) dependent manner. This phenomenon was accompanied by a relocation of AXL to a lateral area between the leading edge and the rear of the polarized migrating cells (Figure 3A,B and Figure S3C–E). The inhibition of AXL downstream effectors such as the PI3K/AKT-Rac1 pathway or SRC kinases did not induce a relocation of AXL at the lateral area in migratory cells (Figure S3F,G).

### 3.4. AXL Inhibition Disrupts Actin Polymerization at the Leading Edge of Migrating Cells

**Figure 4:**
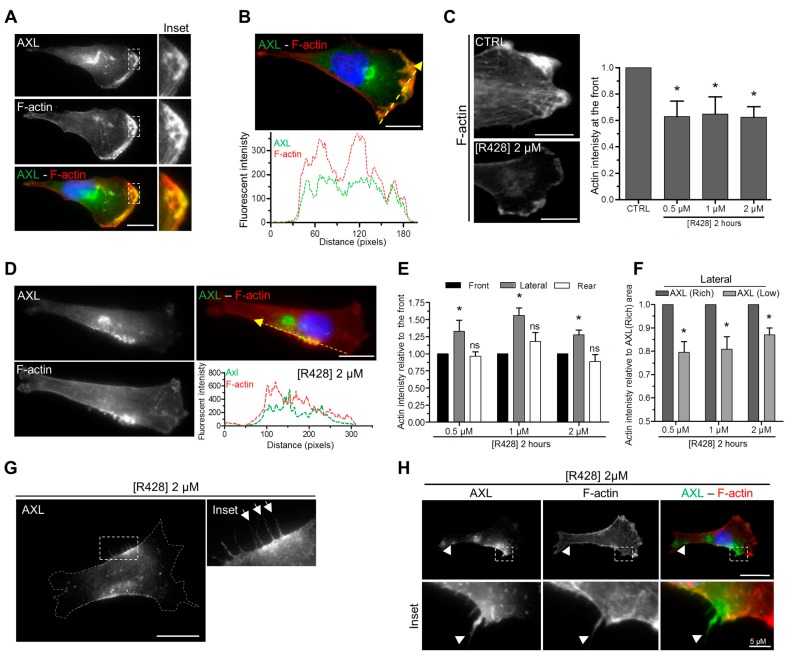
AXL inhibition leads to the loss of actin at the front of migrating cells and its enrichment at the lateral area where AXL relocalizes. (**A**) Hs578t cells stained for AXL (green) and F-actin (red). Inset focus on AXL and F-actin localization at the front of the cell. (**B**) Linescan of AXL (Green) and F-actin (Red) fluorescent intensity at the front of the cell (following the yellow dashed arrow on the top image). (**C**) F-actin intensity at the front of Hs578t cells treated with DMSO (CTRL) or with various concentrations (0.5, 1 and 2 µM) of R428 obtained from 47, 30, 26 and 39 cells showing a relocalization of AXL at the lateral area in three independent experiments, respectively. (* *p* = 0.031; 0.043; 0.014). (**D**) Linescan of AXL and F-actin intensity at the lateral area of the Hs578t cell treated 2 h with 2 µM R428 (following the yellow arrow on the top right image). (**E**) F-actin intensity at the front, at AXL enriched lateral area localization and at the rear of Hs578t cells treated with various concentrations (0.5, 1 and 2 µM) of R428 obtained from respectively 27, 29 and 39 cells in three independent experiments. (ns > 0.05, * *p* = 0.042; 0.012; 0.023). F-actin intensity is normalized to the front to avoid intercellular heterogeneity. (**F**) F-actin intensity at the lateral area (AXL enriched side: AXL(Rich), AXL non-enriched: AXL(Low)) comprised between the front and the rear of Hs578t cells treated with various concentrations (0.5, 1 and 2 µM) of R428 obtained from 27, 29 and 39 cells in three independent experiments, respectively. (* *p* = 0.015; 0.026; 0.016). (**G**,**H**) Representative images of AXL-rich cellular protrusions at the AXL enriched lateral area of Hs578t cells treated with 2 µM R428 for 2 h. The cell is stained for AXL (**G**). The Cell is stained for AXL (Green) and F-actin (Red) (**H**). Arrowheads point to protrusions. Scale bars, 20 µm unless otherwise specified. All graphs represent means and small bars indicate standard deviation.

We observed that AXL co-localizes with actin filaments at the leading edge of migrating cells (Figure 4A,B). As the activation of the small-GTPase protein Rac, acting downstream of AXL, regulates actin polymerization [28,37,59], we next investigated whether AXL invalidation affects actin polymerization in migrating cells. We found that the depletion (Figure S4) or the inhibition (Figure 4C) of AXL significantly reduced the intensity of actin staining at the leading edge of migratory cells. R428 at a dose of 0.5 µM was sufficient to obtain the maximal decrease of actin polymerization at the front of the cells presenting an enrichment of AXL at the lateral area (Figure 4C). Upon AXL inhibition, F-actin became enriched at the lateral area where AXL relocated following its inhibition (Figure 4D–F). At this specific site, we observed protrusions enriched for both AXL and actin (Figure 4G,H).

Our data show that AXL and F-actin co-localized at the leading edge of migratory cells and that AXL participates in actin polymerization at the front.

### 3.5. AXL Controls the Polarized Position of the Golgi Apparatus during Migration

**Figure 5:**
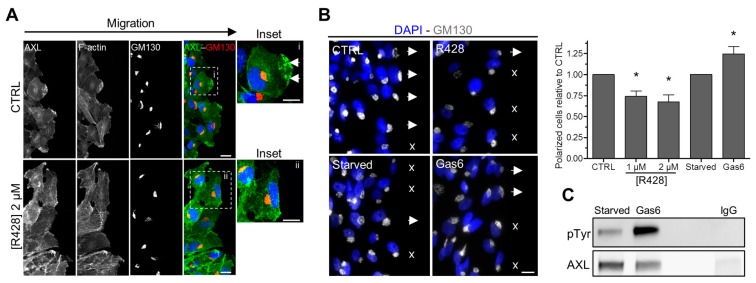
AXL regulates the polarized position of the Golgi apparatus during directed migration. (**A**) Representative images of Hs578t cells treated with DMSO (CTRL) or 2 µM R428 after wound healing (4 h). The cells were stained for F-actin, AXL (Green) and GM130 (Red). Arrowheads point to AXL enrichment in polarized cell. (**B**) Left panel: representative images of Hs578t cells treated with DMSO (CTRL) or 2 µM R428 (R428) or starved (Starved) and then stimulated with 400 ng/mL of Gas6 (Gas6) obtained from 350, 264, 280, 199 and 207 cells in three independent experiments, respectively. The cells were stained for DAPI and GM130. Arrowheads show a polarized Golgi (towards to the wound), crosses show unpolarized Golgi. Right panel: Quantification of cells possessing a polarized Golgi relative to the CTRL condition. The graph represents means and small bars indicate standard deviation (* *p* = 0.021; 0.022; 0.039). (**C**) Gas6 leads to AXL activation. Hs578t cells were starved and then stimulated with 400 ng/mL of Gas6 for 30 min. Phosphorylated active AXL was then detected by western blotting using an anti-phosphotyrosine antibody after AXL immunoprecipitation (as a negative control, IgG instead of AXL antibodies were used with cells treated with DMSO). Scale bars, 20 µm.

The polarized position of the Golgi apparatus, facing the front of migration, has been shown to be crucial in directed migration [60]. We therefore examined whether AXL activity controls the position of the Golgi apparatus in a wound healing experiment. First, we confirmed that AXL was localized at the leading edge of the migrating cells (Figure 2C), facing the wound, in untreated cells and not in R428-treated cells (Figure 5A). Then, we analyzed the position of the Golgi apparatus; the cells being considered polarized when the Golgi apparatus was facing the wound. We found that the cells treated with R428 were less polarized (Figure 5B). Conversely, the incubation of cells with Gas6, which induced AXL phosphorylation (Figure 5C), increased significantly the number of polarized cells (Figure 5B). 

Altogether, we show that AXL contribute to the polarized position of the Golgi apparatus during migration.

### 3.6. AXL-Expressing Tumor Cells Are Preferentially Located in Contact with the Stroma in Human TNBC

**Figure 6:**
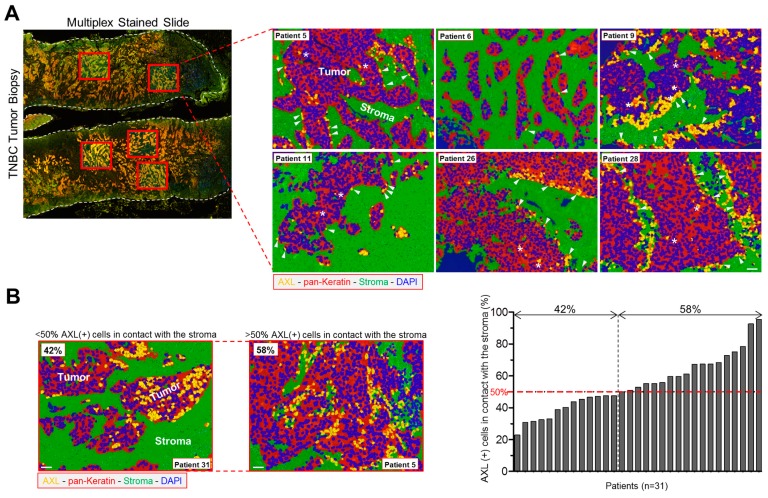
AXL expressing tumor cells are preferentially localized in contact with the stroma in untreated aggressive TNBC biopsies. (**A**) Left panel: image of a multiplex stained slide from a human TNBC sample with selected areas for analysis (red boxes). The white dashed lines delineate the tumor sample. Right panel: representative images obtained from six different TNBC patients showing AXL-positive cells at the contact with the stroma. Staining: AXL (yellow), pan-Keratin-AE1/AE3 (red) and DAPI (blue). The area that was negative for cytokeratin was considered as stroma and stained in green. Arrowheads point to AXL-positive cells in contact with the stroma, stars point to AXL-positive cells that are not close to the stroma. (**B**) Left panel: representative images from two TNBC patients: AXL (yellow), pan-Keratin-AE1/AE3 (red), DAPI (blue), stroma (green). Right panel: AXL-expressing (AXL (+)) cells (%) in contact with the stroma in biopsies obtained from 31 TNBC patients. Patient’s number: number of regions counted/number of total cells counted; P1: 3/159; P2: 4/17; P3: 6/43; P4: 7/27; P5: 8/579; P6: 9/216; P7: 8/130; P8: 3/57; P9: 4/623; P10: 7/280; P11: 2/386; P12: 4/166; P13: 3/96; P14: 5/199; P15: 2/304; P16: 5/75; P17: 6/121; P18: 6/143; P19: 7/70; P20: 7/438; P21: 3/204; P22: 4/133; P23: 9/190; P24: 4/290; P25: 3/91; P26: 4/174; P27: 5/160; P28: 4/227; P29: 4/205; P30: 3/158; P31: 2/360. Scale bars, 50 µm.

We examined the expression of AXL at the protein level in 31 human TNBC samples obtained from patients before receiving neoadjuvant treatment. These patients did not reach pathologic complete response after chemotherapies; hence we focused on a cohort of chemo-resistant TNBC (Table S1). We performed a multiplexed IHC experiment using AXL and pan-keratin antibodies. Keratin staining allowed us to discriminate cancer cells (keratin-positive) from the stroma (keratin-negative) and to explore the proximity of the cancer cells to the tumor microenvironment (Figure 6A,B). We observed a high heterogeneity of AXL staining in these samples (Figure 6A,B). Within a tumor, not all the tumor cells expressed AXL, showing intra-tumor heterogeneity of AXL staining. Importantly, we found that the majority of the AXL-expressing cells (>50%) were in contact with the stroma in 58% of our 31 TNBC samples (Figure 6A,B).

These data highlight inter- and intra-tumor heterogeneity of AXL expression as well as a preferential localization of AXL-positive tumor cells at the proximity of the stroma in therapy-naïve TNBC samples with clinically proven features of chemo-resistance. 

## 4. Discussion

TNBC is the breast cancer subtype associated with the worst prognosis due to high percentage of relapse and metastases following treatment. TNBC encompasses a heterogeneous group including tumors with a mesenchymal phenotype [3,6]. EMT is a crucial process during normal development and contributes to invasion and the formation of metastases in human tumors [11,12,13,14,15,16,17,37,61,62]. AXL is a protein kinase modulating EMT, which is involved in cell invasion and resistance to therapies [23,28,31,32,37,63,64]. 

We focused our study on mesenchymal TNBC cells and found that they express higher levels of AXL compared to luminal cells as previously reported [38,49,65,66]. The expression of AXL was variable among the mesenchymal TNBC cell lines, some expressing low levels (BT549, MDA-MB-157 and MDA-MB-436), and some expressing high levels (MDA-MB-231 and Hs578T), in agreement with previous reports [38,49,67,68]. We found that AXL invalidation had little/no effect on cell viability/proliferation but impairs cell migration speed, as previously shown [33,47,49,55,56,57,58]. These results were also in concordance with an in vivo study showing that AXL is not involved in tumor growth but regulates the dissemination of cancer cells and the development of metastases [69].

In this study, we further characterized the function of AXL in migration and we provided evidence that AXL controls directed migration. Indeed, its depletion or its inhibition impaired the directionality of the two studied mesenchymal TNBC cell lines (MDA-MB-231 and Hs578T). Using a cell biology approach, we then tried to understand by which molecular mechanisms AXL could affect directed migration. Mainly due to poor quality antibodies that are commercially available for immunofluorescence analyses, very few studies have addressed the intracellular localization of AXL [33]. Here, using two different validated anti-AXL antibodies, we show that AXL had a polarized localization in migrating mesenchymal TNBC cells being mainly present at the leading edge and at the Golgi apparatus. Although its localization at the front was not surprising due to its established function in migration and invasion, its localization at the Golgi apparatus was more unexpected.

To our knowledge, the presence of AXL at the lamellipodia of breast cancer polarized cells toward the scratch wound has been documented only in MDA-MB-231 cells [33]. Another study indicates that the protrusions, which are induced when MDA-MB-231 cells are stimulated by EGF, are reduced when the cells are depleted for AXL [26].

This is the first time that AXL is described to be localized at the Golgi apparatus. Although not discussed by the authors, a perinuclear AXL staining has been observed in migratory MCF10A cells [33]. Interestingly, we observed AXL positive-vesicles between the Golgi apparatus and the migrating front, suggesting a trafficking of AXL between these two compartments. It is possible that AXL is specifically transported to the leading edge of migratory cells via these vesicles. Moreover, once activated, AXL could be endocytosed to the Golgi apparatus before recycling as shown for other transmembrane receptors such as EGFR [70]. These hypotheses could be verified by live-imaging with cells expressing ectopically a fluorescent-tagged AXL. Noteworthy, AXL has been shown recently to mediate the anterograde trafficking of lysosomes (Lamp1-positive vesicles) in esophageal adenocarcinoma cells [58], regulating the secretion of cathepsin B [58], which is involved in matrix degradation and invasion [71,72]. Altogether, these results suggest that AXL may regulate migration and invasion by controlling vesicle trafficking. Further experiments are required to decipher the molecular mechanisms by which AXL acts on membrane trafficking.

Directed migration of mesenchymal cells involves polarized and dynamic architecture of the actin cytoskeleton [60]. We observed that AXL colocalizes with F-actin at the leading edge of migrating cells. The inhibition of AXL with the specific inhibitor R428 decreases F-actin intensity at the front of migrating cells suggesting that AXL controls actin polymerization at that site. We did not observe a complete decrease of actin polymerization at the front upon Axl inhibition suggesting that actin polymerization did not rely solely on Axl activity. Interestingly, AXL inhibition displaced AXL in migrating cells from the leading edge to a lateral area localized between the front and the rear of the cells. This suggests that the kinase activity of AXL was required for its presence at the leading edge during migration. Following treatment with R428, F-actin became enriched at the specific lateral area where AXL was mislocalized. The kinase activity of AXL was not by itself required for F-actin polymerization because F-actin polymerization was still stimulated at the lateral area of R428-treated cells where AXL relocalizes.

We also reported that AXL inhibition affected the polarized location of the Golgi apparatus in a wound healing assay, indicating that AXL activity was required for this process. The molecular mechanisms by which AXL controls the polarity of the Golgi apparatus could be possibly though the phosphorylation of specific substrates at and/or close to the Golgi as shown for polarity proteins such as Par6α [73]. 

The presence of filaments (or membrane extensions) containing both AXL and F-actin observed upon AXL inhibition at the lateral side of the migrating cells, could also explain why AXL inhibition disrupts directed migration. Regarding protrusions, it has been recently shown that AXL invalidation enhances invadopodia formation in melanoma cells [74]. Many studies have reported a role for AXL in invasion in various cellular models, including breast cancer cells [23,28,31,64], but whether it occurs through controlling invadopodia formation remains to be investigated.

We explored the localization of AXL in our cohort of chemo-resistant TNBC obtained before neoadjuvant treatment. In these samples, we detected both inter- and intra-tumor heterogeneity regarding the number of tumor cells expressing AXL. We also observed that AXL was mainly expressed by tumor cells in contact with the stroma. A recent study shows that AXL expression is higher in collagen-I rich microenvironment [75] suggesting that the extracellular matrix could have an impact in AXL expression. In breast tumors, Gas6 is highly expressed in pre-invasive lesions associated with increased infiltrating macrophages [76]. Gas6 produced by macrophages is essential for the transition of premalignant to invasive breast cancer [76]. Another study shows that AXL-expressing TNBC cells can polarize macrophages towards an activated phenotype producing pro-tumorigenic chemokines and cytokines [66]. The authors further show that AXL inhibition decreases the activity of these activated macrophages reducing cancer cell invasiveness and restoring drug sensitivity of cancer cells [66]. Cancer-associated fibroblasts can also produce Gas6 following therapy leading to the migration of AXL-positive lung cancer cells [77]. Collectively, the contact with extracellular matrix may lead to AXL overexpression in cancer cells and Gas6, secreted by some stromal cells, could induce the polarization of the Golgi toward the microenvironment allowing the escape of tumor cells.

In conclusion, we found that AXL is polarized in migrating cells and that AXL plays a role in directed migration most likely by controlling the localization of AXL and F-actin at the leading edge and Golgi apparatus polarization. AXL could be a powerful actor of the ability of cancer cells to escape the tumor core through its expression in cells at the contact with the stroma and its stimulation by Gas6 secreted in the tumor microenvironment.

## Supplementary Materials

The Table S1 and Figures S1–S4 are available online at https://www.mdpi.com/2073-4409/9/1/247/s1.

## Abbreviations

AXL	AXL receptor tyrosine kinase
DMSO	Dimethyl Sulfoxide
DTT	1:4-dithio-dl-threitol
EGFR	Epidermal Growth Factor Receptor 1
EMT	Epithelial-to-Mesenchymal Transition
ER	Estrogen Receptor
F-actin	Filament of actin
FBS	Fetal Bovine Serum
GAS6	Growth Arrest Specific protein 6
IHC	Immunohistochemistry
INCa	French National Cancer Institute
IF	Immunofluorescence
IP	Immunoprecipitation
Lamp1	Lysosomal Associated Membrane Protein 1
MERTK	MER receptor Tyrosine Kinase
PR	Progesterone Receptor
P/S	Penicillin and streptomycin
Rac	Rac family of small GTPases
RTK	Receptor Tyrosine Kinases
TNBC	Triple Negative Breast Cancer
TYRO3	TYRO3 receptor tyrosine kinase

## References

[1] Denkert C., Liedtke C., Tutt A., von Minckwitz G. (2016). Molecular alterations in triple-negative breast cancer-the road to new treatment strategies. Lancet.

[2] Jitariu A.A., Cimpean A.M., Ribatti D., Raica M. (2017). Triple negative breast cancer: The kiss of death. Oncotarget.

[3] Lehmann B.D., Jovanovic B., Chen X., Estrada M.V., Johnson K.N., Shyr Y., Moses H.L., Sanders M.E., Pietenpol J.A. (2016). Refinement of Triple-Negative Breast Cancer Molecular Subtypes: Implications for Neoadjuvant Chemotherapy Selection. PLoS ONE.

[4] Karaayvaz M., Cristea S., Gillespie S.M., Patel A.P., Mylvaganam R., Luo C.C., Specht M.C., Bernstein B.E., Michor F., Ellisen L.W. (2018). Unravelling subclonal heterogeneity and aggressive disease states in TNBC through single-cell RNA-seq. Nat. Commun..

[5] Wang D.Y., Jiang Z., Ben-David Y., Woodgett J.R., Zacksenhaus E. (2019). Molecular stratification within triple-negative breast cancer subtypes. Sci. Rep..

[6] Lehmann B.D., Bauer J.A., Chen X., Sanders M.E., Chakravarthy A.B., Shyr Y., Pietenpol J.A. (2011). Identification of human triple-negative breast cancer subtypes and preclinical models for selection of targeted therapies. J. Clin. Investig..

[7] Ahn S.G., Kim S.J., Kim C., Jeong J. (2016). Molecular Classification of Triple-Negative Breast Cancer. J. Breast Cancer.

[8] Fedele M., Cerchia L., Chiappetta G. (2017). The Epithelial-to-Mesenchymal Transition in Breast Cancer: Focus on Basal-Like Carcinomas. Cancers.

[9] Gu G., Dustin D., Fuqua S.A. (2016). Targeted therapy for breast cancer and molecular mechanisms of resistance to treatment. Curr. Opin. Pharm..

[10] Yang F., Wang Y., Li Q., Cao L., Sun Z., Jin J., Fang H., Zhu A., Li Y., Zhang W. (2017). Intratumor heterogeneity predicts metastasis of triple-negative breast cancer. Carcinogenesis.

[11] Antony J., Thiery J.P., Huang R.Y. (2019). Epithelial-to-mesenchymal transition: Lessons from development, insights into cancer and the potential of EMT-subtype based therapeutic intervention. Phys. Biol..

[12] Lamouille S., Xu J., Derynck R. (2014). Molecular mechanisms of epithelial-mesenchymal transition. Nat. Rev. Mol. Cell Biol..

[13] Dongre A., Weinberg R.A. (2019). New insights into the mechanisms of epithelial-mesenchymal transition and implications for cancer. Nat. Rev. Mol. Cell Biol..

[14] Puisieux A., Pommier R.M., Morel A.P., Lavial F. (2018). Cellular Pliancy and the Multistep Process of Tumorigenesis. Cancer Cell.

[15] Pastushenko I., Blanpain C. (2019). EMT Transition States during Tumor Progression and Metastasis. Trends Cell Biol..

[16] Chaffer C.L., San Juan B.P., Lim E., Weinberg R.A. (2016). EMT, cell plasticity and metastasis. Cancer Metastasis Rev..

[17] Lu W., Kang Y. (2019). Epithelial-Mesenchymal Plasticity in Cancer Progression and Metastasis. Dev. Cell.

[18] Voon D.C., Huang R.Y., Jackson R.A., Thiery J.P. (2017). The EMT spectrum and therapeutic opportunities. Mol. Oncol..

[19] Santamaria P.G., Moreno-Bueno G., Cano A. (2019). Contribution of Epithelial Plasticity to Therapy Resistance. J. Clin. Med..

[20] Shibue T., Weinberg R.A. (2017). EMT, CSCs, and drug resistance: The mechanistic link and clinical implications. Nat. Rev. Clin. Oncol..

[21] Marcucci F., Stassi G., De Maria R. (2016). Epithelial-mesenchymal transition: A new target in anticancer drug discovery. Nat. Rev. Drug Discov..

[22] O’Bryan J.P., Frye R.A., Cogswell P.C., Neubauer A., Kitch B., Prokop C., Espinosa R., Le Beau M.M., Earp H.S., Liu E.T. (1991). axl, a transforming gene isolated from primary human myeloid leukemia cells, encodes a novel receptor tyrosine kinase. Mol. Cell. Biol..

[23] Graham D.K., DeRyckere D., Davies K.D., Earp H.S. (2014). The TAM family: Phosphatidylserine sensing receptor tyrosine kinases gone awry in cancer. Nat. Rev. Cancer.

[24] Stitt T.N., Conn G., Gore M., Lai C., Bruno J., Radziejewski C., Mattsson K., Fisher J., Gies D.R., Jones P.F. (1995). The anticoagulation factor protein S and its relative, Gas6, are ligands for the Tyro 3/Axl family of receptor tyrosine kinases. Cell.

[25] Varnum B.C., Young C., Elliott G., Garcia A., Bartley T.D., Fridell Y.W., Hunt R.W., Trail G., Clogston C., Toso R.J. (1995). Axl receptor tyrosine kinase stimulated by the vitamin K-dependent protein encoded by growth-arrest-specific gene 6. Nature.

[26] Meyer A.S., Miller M.A., Gertler F.B., Lauffenburger D.A. (2013). The receptor AXL diversifies EGFR signaling and limits the response to EGFR-targeted inhibitors in triple-negative breast cancer cells. Sci. Signal..

[27] Rankin E.B., Giaccia A.J. (2016). The Receptor Tyrosine Kinase AXL in Cancer Progression. Cancers.

[28] Schoumacher M., Burbridge M. (2017). Key Roles of AXL and MER Receptor Tyrosine Kinases in Resistance to Multiple Anticancer Therapies. Curr. Oncol Rep..

[29] Gjerdrum C., Tiron C., Hoiby T., Stefansson I., Haugen H., Sandal T., Collett K., Li S., McCormack E., Gjertsen B.T. (2010). Axl is an essential epithelial-to-mesenchymal transition-induced regulator of breast cancer metastasis and patient survival. Proc. Natl. Acad. Sci. USA.

[30] Asiedu M.K., Beauchamp-Perez F.D., Ingle J.N., Behrens M.D., Radisky D.C., Knutson K.L. (2014). AXL induces epithelial-to-mesenchymal transition and regulates the function of breast cancer stem cells. Oncogene.

[31] Paccez J.D., Vogelsang M., Parker M.I., Zerbini L.F. (2014). The receptor tyrosine kinase Axl in cancer: Biological functions and therapeutic implications. Int. J. Cancer.

[32] Wu X., Liu X., Koul S., Lee C.Y., Zhang Z., Halmos B. (2014). AXL kinase as a novel target for cancer therapy. Oncotarget.

[33] Vuoriluoto K., Haugen H., Kiviluoto S., Mpindi J.P., Nevo J., Gjerdrum C., Tiron C., Lorens J.B., Ivaska J. (2011). Vimentin regulates EMT induction by Slug and oncogenic H-Ras and migration by governing Axl expression in breast cancer. Oncogene.

[34] Vouri M., Hafizi S. (2017). TAM Receptor Tyrosine Kinases in Cancer Drug Resistance. Cancer Res..

[35] Scaltriti M., Elkabets M., Baselga J. (2016). Molecular Pathways: AXL, a Membrane Receptor Mediator of Resistance to Therapy. Clin. Cancer Res..

[36] Goyette M.A., Duhamel S., Aubert L., Pelletier A., Savage P., Thibault M.P., Johnson R.M., Carmeliet P., Basik M., Gaboury L. (2018). The Receptor Tyrosine Kinase AXL Is Required at Multiple Steps of the Metastatic Cascade during HER2-Positive Breast Cancer Progression. Cell Rep..

[37] Antony J., Huang R.Y. (2017). AXL-Driven EMT State as a Targetable Conduit in Cancer. Cancer Res..

[38] Wilson C., Ye X., Pham T., Lin E., Chan S., McNamara E., Neve R.M., Belmont L., Koeppen H., Yauch R.L. (2014). AXL inhibition sensitizes mesenchymal cancer cells to antimitotic drugs. Cancer Res..

[39] Liu L., Greger J., Shi H., Liu Y., Greshock J., Annan R., Halsey W., Sathe G.M., Martin A.M., Gilmer T.M. (2009). Novel mechanism of lapatinib resistance in HER2-positive breast tumor cells: Activation of AXL. Cancer Res..

[40] Zhang Z., Lee J.C., Lin L., Olivas V., Au V., LaFramboise T., Abdel-Rahman M., Wang X., Levine A.D., Rho J.K. (2012). Activation of the AXL kinase causes resistance to EGFR-targeted therapy in lung cancer. Nat. Genet..

[41] Byers L.A., Diao L., Wang J., Saintigny P., Girard L., Peyton M., Shen L., Fan Y., Giri U., Tumula P.K. (2013). An epithelial-mesenchymal transition gene signature predicts resistance to EGFR and PI3K inhibitors and identifies Axl as a therapeutic target for overcoming EGFR inhibitor resistance. Clin. Cancer Res..

[42] Rankin E.B., Fuh K.C., Taylor T.E., Krieg A.J., Musser M., Yuan J., Wei K., Kuo C.J., Longacre T.A., Giaccia A.J. (2010). AXL is an essential factor and therapeutic target for metastatic ovarian cancer. Cancer Res..

[43] Creedon H., Gomez-Cuadrado L., Tarnauskaite Z., Balla J., Canel M., MacLeod K.G., Serrels B., Fraser C., Unciti-Broceta A., Tracey N. (2016). Identification of novel pathways linking epithelial-to-mesenchymal transition with resistance to HER2-targeted therapy. Oncotarget.

[44] Hong C.C., Lay J.D., Huang J.S., Cheng A.L., Tang J.L., Lin M.T., Lai G.M., Chuang S.E. (2008). Receptor tyrosine kinase AXL is induced by chemotherapy drugs and overexpression of AXL confers drug resistance in acute myeloid leukemia. Cancer Lett..

[45] Kurokawa M., Ise N., Omi K., Goishi K., Higashiyama S. (2013). Cisplatin influences acquisition of resistance to molecular-targeted agents through epithelial-mesenchymal transition-like changes. Cancer Sci..

[46] Shen Y., Chen X., He J., Liao D., Zu X. (2018). Axl inhibitors as novel cancer therapeutic agents. Life Sci..

[47] Holland S.J., Pan A., Franci C., Hu Y., Chang B., Li W., Duan M., Torneros A., Yu J., Heckrodt T.J. (2010). R428, a selective small molecule inhibitor of Axl kinase, blocks tumor spread and prolongs survival in models of metastatic breast cancer. Cancer Res..

[48] Vouri M., An Q., Birt M., Pilkington G.J., Hafizi S. (2015). Small molecule inhibition of Axl receptor tyrosine kinase potently suppresses multiple malignant properties of glioma cells. Oncotarget.

[49] Zhang Y.X., Knyazev P.G., Cheburkin Y.V., Sharma K., Knyazev Y.P., Orfi L., Szabadkai I., Daub H., Keri G., Ullrich A. (2008). AXL is a potential target for therapeutic intervention in breast cancer progression. Cancer Res..

[50] D’Alfonso T.M., Hannah J., Chen Z., Liu Y., Zhou P., Shin S.J. (2014). Axl receptor tyrosine kinase expression in breast cancer. J. Clin. Pathol..

[51] Vinet M., Suresh S., Maire V., Monchecourt C., Nemati F., Lesage L., Pierre F., Ye M., Lescure A., Brisson A. (2019). Protein arginine methyltransferase 5: A novel therapeutic target for triple-negative breast cancers. Cancer Med..

[52] Maubant S., Tahtouh T., Brisson A., Maire V., Némati F., Tesson B., Ye M., Rigaill G., Noizet M., Dumont A. (2018). LRP5 regulates the expression of STK40, a new potential target in triple-negative breast cancers. Oncotarget.

[53] Baldeyron C., Brisson A., Tesson B., Nemati F., Koundrioukoff S., Saliba E., De Koning L., Martel E., Ye M., Rigaill G. (2015). TIPIN depletion leads to apoptosis in breast cancer cells. Mol. Oncol..

[54] Maire V., Mahmood F., Rigaill G., Ye M., Brisson A., Nemati F., Gentien D., Tucker G.C., Roman-Roman S., Dubois T. (2019). LRP8 is overexpressed in estrogen-negative breast cancers and a potential target for these tumors. Cancer Med..

[55] Holland S.J., Powell M.J., Franci C., Chan E.W., Friera A.M., Atchison R.E., McLaughlin J., Swift S.E., Pali E.S., Yam G. (2005). Multiple roles for the receptor tyrosine kinase axl in tumor formation. Cancer Res..

[56] McDaniel N.K., Cummings C.T., Iida M., Hulse J., Pearson H.E., Vasileiadi E., Parker R.E., Orbuch R.A., Ondracek O.J., Welke N.B. (2018). MERTK Mediates Intrinsic and Adaptive Resistance to AXL-targeting Agents. Mol. Cancer.

[57] Uribe D.J., Mandell E.K., Watson A., Martinez J.D., Leighton J.A., Ghosh S., Rothlin C.V. (2017). The receptor tyrosine kinase AXL promotes migration and invasion in colorectal cancer. PLoS ONE.

[58] Maacha S., Hong J., von Lersner A., Zijlstra A., Belkhiri A. (2018). AXL Mediates Esophageal Adenocarcinoma Cell Invasion through Regulation of Extracellular Acidification and Lysosome Trafficking. Neoplasia.

[59] Ridley A.J. (2015). Rho GTPase signalling in cell migration. Curr. Opin. Cell Biol..

[60] Bear J.E., Haugh J.M. (2014). Directed migration of mesenchymal cells: Where signaling and the cytoskeleton meet. Curr. Opin. Cell Biol..

[61] Pearson G.W. (2019). Control of Invasion by Epithelial-to-Mesenchymal Transition Programs during Metastasis. J. Clin. Med..

[62] Thiery J.P., Acloque H., Huang R.Y., Nieto M.A. (2009). Epithelial-mesenchymal transitions in development and disease. Cell.

[63] Axelrod H., Pienta K.J. (2014). Axl as a mediator of cellular growth and survival. Oncotarget.

[64] Gay C.M., Balaji K., Byers L.A. (2017). Giving AXL the axe: Targeting AXL in human malignancy. Br. J. Cancer.

[65] Li Y., Jia L., Liu C., Gong Y., Ren D., Wang N., Zhang X., Zhao Y. (2015). Axl as a downstream effector of TGF-beta1 via PI3K/Akt-PAK1 signaling pathway promotes tumor invasion and chemoresistance in breast carcinoma. Tumour Biol..

[66] Bottai G., Raschioni C., Szekely B., Di Tommaso L., Szasz A.M., Losurdo A., Gyorffy B., Acs B., Torrisi R., Karachaliou N. (2016). AXL-associated tumor inflammation as a poor prognostic signature in chemotherapy-treated triple-negative breast cancer patients. Npj Breast Cancer.

[67] Wang C., Jin H., Wang N., Fan S., Wang Y., Zhang Y., Wei L., Tao X., Gu D., Zhao F. (2016). Gas6/Axl Axis Contributes to Chemoresistance and Metastasis in Breast Cancer through Akt/GSK-3beta/beta-catenin Signaling. Theranostics.

[68] Leconet W., Chentouf M., du Manoir S., Chevalier C., Sirvent A., Ait-Arsa I., Busson M., Jarlier M., Radosevic-Robin N., Theillet C. (2017). Therapeutic Activity of Anti-AXL Antibody against Triple-Negative Breast Cancer Patient-Derived Xenografts and Metastasis. Clin. Cancer Res..

[69] Del Pozo Martin Y., Park D., Ramachandran A., Ombrato L., Calvo F., Chakravarty P., Spencer-Dene B., Derzsi S., Hill C.S., Sahai E. (2015). Mesenchymal Cancer Cell-Stroma Crosstalk Promotes Niche Activation, Epithelial Reversion, and Metastatic Colonization. Cell Rep..

[70] Ye Q.H., Zhu W.W., Zhang J.B., Qin Y., Lu M., Lin G.L., Guo L., Zhang B., Lin Z.H., Roessler S. (2016). GOLM1 Modulates EGFR/RTK Cell-Surface Recycling to Drive Hepatocellular Carcinoma Metastasis. Cancer Cell.

[71] Gondi C.S., Rao J.S. (2013). Cathepsin B as a cancer target. Expert Opin. Ther. Targets.

[72] Chen B.J., Tang Y.J., Tang Y.L., Liang X.H. (2019). What makes cells move: Requirements and obstacles for leader cells in collective invasion. Exp. Cell Res..

[73] Pallesi-Pocachard E., Bazellieres E., Viallat-Lieutaud A., Delgrossi M.H., Barthelemy-Requin M., Le Bivic A., Massey-Harroche D. (2016). Hook2, a microtubule-binding protein, interacts with Par6alpha and controls centrosome orientation during polarized cell migration. Sci. Rep..

[74] Revach O.Y., Sandler O., Samuels Y., Geiger B. (2019). Cross-Talk between Receptor Tyrosine Kinases AXL and ERBB3 Regulates Invadopodia Formation in Melanoma Cells. Cancer Res..

[75] Jokela T.A., Engelsen A.S.T., Rybicka A., Pelissier Vatter F.A., Garbe J.C., Miyano M., Tiron C., Ferariu D., Akslen L.A., Stampfer M.R. (2018). Microenvironment-Induced Non-sporadic Expression of the AXL and cKIT Receptors Are Related to Epithelial Plasticity and Drug Resistance. Front. Cell Dev. Biol..

[76] Gomes A.M., Carron E.C., Mills K.L., Dow A.M., Gray Z., Fecca C.R., Lakey M.A., Carmeliet P., Kittrell F., Medina D. (2019). Stromal Gas6 promotes the progression of premalignant mammary cells. Oncogene.

[77] Kanzaki R., Naito H., Kise K., Takara K., Eino D., Minami M., Shintani Y., Funaki S., Kawamura T., Kimura T. (2017). Gas6 derived from cancer-associated fibroblasts promotes migration of Axl-expressing lung cancer cells during chemotherapy. Sci. Rep..

