# Supplementary material for: AXL Controls Directed Migration of Mesenchymal Triple-Negative Breast Cancer Cells
Source: Cells. 2020 Jan 19;9(1):247. doi: 10.3390/cells9010247 (PMC7016818; doi:10.3390/cells9010247)
Supplement: Supplementary file 1 [file cells-09-00247-s001.pdf]

cells

MDPI

AXL controls directed migration of mesenchymal triple-negative breast cancer cells

Olivier Zajac[1], Renaud Leclere[2], André Nicolas[2], Didier Meseure[2], Caterina Marchiò[3,4,^], Anne Vincent-Salomon[4], Sergio Roman-Roman[5], Marie Schoumacher[6] and Thierry Dubois[1,*]

[1] Institut Curie, PSL Research University, Translational Research Department, Breast Cancer Biology Group, 75005 Paris, France; olivier.zajac@curie.fr (O.Z.); thierry.dubois@curie.fr (T.D.)
[2] Institut Curie, PSL Research University, Platform of Investigative Pathology, Department of Pathology, 75005 Paris, France; renaud.leclere@curie.fr (R.L.); andre.nicolas@curie.fr (A.N.); didier.meseure@curie.fr (D.M.)
[3] Department of Medical Sciences, University of Turin, Via Verdi 8, Italy; caterina.marchio@unito.it (C.M.)
[4] Institut Curie, PSL Research University, Department of Pathology, 75005 Paris, France ; caterina.marchio@unito.it (C.M.) ; anne.salomon@curie.fr (A.V.S.)
[5] Institut Curie, PSL Research University, Translational Research Department, 75005 Paris, France; sergio.roman-roman@curie.fr (S.R.R.)
[6] Center for Therapeutic Innovation in Oncology, Institut de Recherches Internationales SERVIER, 92284 Suresnes, France; marie.schoumacher@servier.com (M.S.)

[^] Current affiliation : Candiolo Cancer Institute, FPO-IRCCS, Candiolo, Italy

[*] Correspondence: thierry.dubois@curie.fr (T.D.); Tel.: +33-156246250

Table S1 - Supplementary figures 1 - 4

Patient n°	GENDER	Breast cancer Subtype	Neoadjuvant treatment	Residual disease	RCB	Axl-positive cells in contact to stroma (%)
1	F	TNBC	Fec100 + docetaxel	+	ND	67.3
2	F	TNBC	Fec100 + docetaxel	+	III	52.9
3	F	TNBC	Fec100 + docetaxel	+	II	55.8
4	F	TNBC	Fec75 + docetaxel	+	III	92.6
5	F	TNBC	Fec100 + docetaxel	+	II	72.9
6	F	TNBC	AC + paclitaxel	+	III	55.1
7	F	TNBC	AC + paclitaxel	+	III	95.4
8	F	TNBC	AC + paclitaxel	+	II	59.6
9	F	TNBC	Fec75 + docetaxel	+	II	59.6
10	F	TNBC	Fec100 + docetaxel	+	ND	67.1
11	F	TNBC	AC + docetaxel	+	ND	45.3
12	F	TNBC	Fec100 + docetaxel	+	ND	32.5
13	F	TNBC	Fec100 + docetaxel	+	II	43.7
14	F	TNBC	Cyclophosphamide & epirubicin + docetaxel	+	ND	40.2
15	F	TNBC	Fec100 + docetaxel	+	II	61.2
16	F	TNBC	Fec100 + docetaxel	+	II	75.0
17	F	TNBC	Fec100 + docetaxel	+	ND	31.4
18	F	TNBC	Fec100 + docetaxel	+	III	47.6
19	F	TNBC	Fec100 + docetaxel	+	ND	47.1
20	F	TNBC	Cyclophosphamide & epirubicin + docetaxel	+	ND	46.6
21	F	TNBC	AC + paclitaxel	+	II	51.0
22	F	TNBC	AC + paclitaxel	+	II	30.8
23	F	TNBC	Fec100 + docetaxel	+	ND	78.4
24	F	TNBC	Fec100 + docetaxel	+	III	55.2
25	F	TNBC	AC + paclitaxel	+	II	33.0
26	F	TNBC	AC + paclitaxel	+	II	50.0
27	F	TNBC	AC + paclitaxel	+	II	47.5
28	F	TNBC	Fec100 + docetaxel	+	I	67.4
29	F	TNBC	Fec100 + docetaxel	+	II	22.9
30	F	TNBC	Fec100 + docetaxel	+	II	68.4
31	F	TNBC	Fec100 + docetaxel	+	II	38.9

Table S1 | Human patient samples analyzed for Axl staining. Thirty-one female TNBC patients were selected on the criteria that they displayed a residual disease after neoadjuvant treatment. The Residual Cancer Burden (RCB) index (I-II-III) after neoadjuvant treatment is associated with risk for relapse and mortality (higher is the number, higher is the risk). For each patient, the percentage of Axl-positive tumor cells in contact to the stroma is mentioned. ND, not determined.

Supplementary fig.1

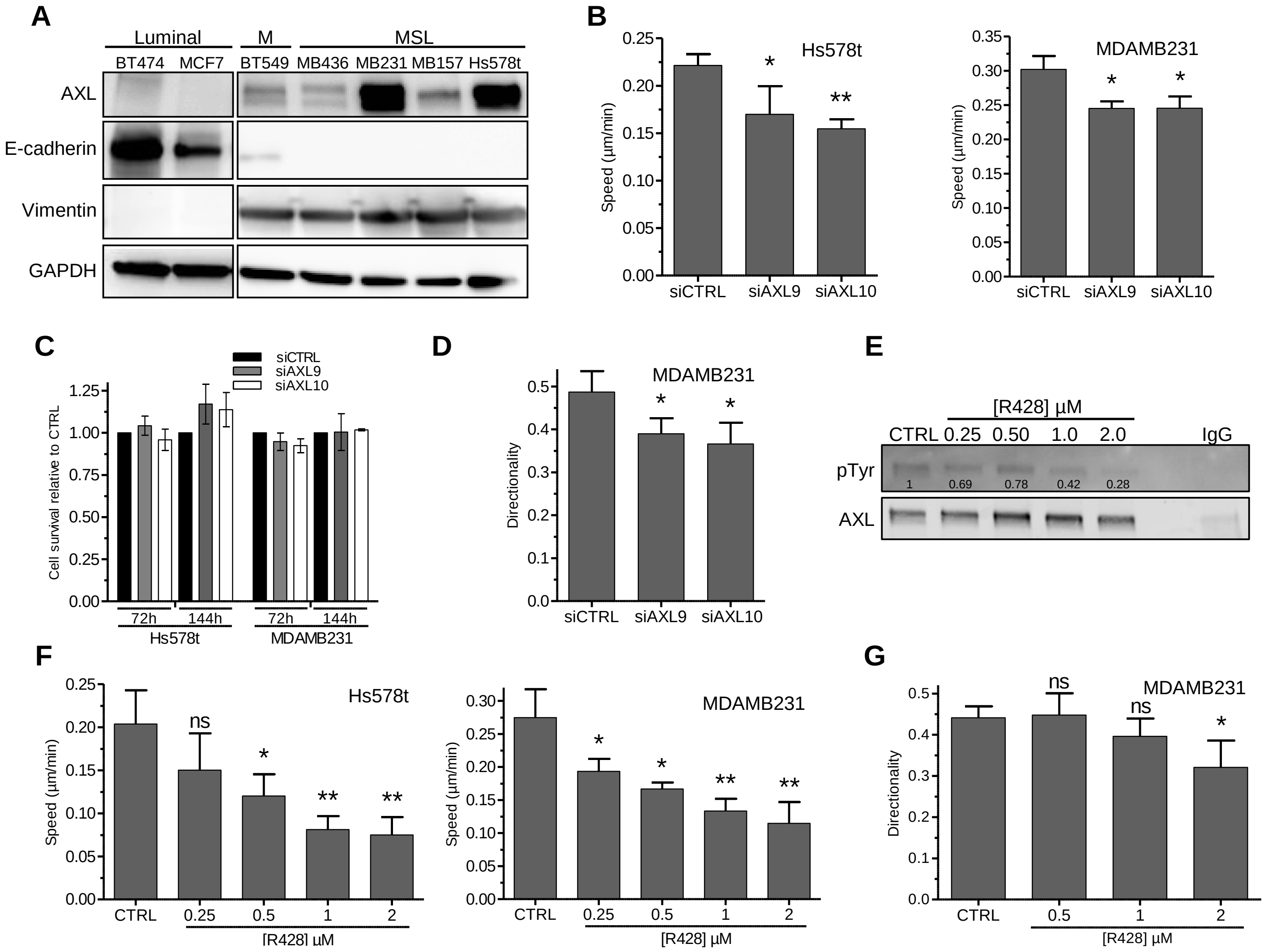

Supplementary fig.1 | (A) AXL, E-cadherin and Vimentin protein expression by western-blotting in seven breast cancer cell lines (M: Mesenchymal; MSL: Mesenchymal Stem Like, MB436: MDA-MB-436, MB231: MDA-MB-231, MB157: MDA-MB-157). GAPDH was used as a loading control. **(B)** Speed of Hs578t (Left) and MDA-MB-231 (Right) cells during random migration, three days after transfection with CTRL, AXL9 or AXL10 siRNA obtained from 110, 100 and 113 cells in 3 independent experiments for Hs578t (*p = 0.049, **p = 0.0017) and from 121, 93 and 97 cells in 3 independent experiments for MDA-MB-231, respectively (*p = 0.011 - 0.019). **(C)** Cell survival (CelltiterGlo Assay) 72h and 144h post-transfection with CTRL, AXL9 and AXL10 siRNA. **(D)** Evaluation of the directionality of MDA-MB-231 cells three days after transfection with CTRL, AXL9 and AXL10 siRNA obtained from respectively 155, 123 and 126 cells in 4 independent experiments (*p = 0.018 - 0.013). **(E)** MDA-MB-231 cells were cultured with serum and treated with DMSO (CTRL) or various concentrations (0.25, 0.5, 1 or 2 µM) of R428 for 6 h. Basal phosphorylated active AXL was then detected by western blotting using an anti-phosphotyrosine antibody after AXL immunoprecipitation. As a negative control, IgG instead of AXL antibodies were used with cells treated with DMSO. **(F)** Speed of Hs578t (Left) and MDA-MB-231 (Right) cells during random migration after treatment with DMSO (CTRL) or various concentrations (0.25, 0.5, 1 or 2 µM) of R428 obtained from respectively 129, 124, 125 ,110 and 99 cells in 4 independent experiments for the Hs578t (ns > 0.05, *p = 0.0117, **p = 0.0012 - 0.0012) and from 106, 116, 104, 127 and 85 cells in 3 independent experiments for the MDA-MB-231 (*p = 0.039 - 0.013, **p = 0.0062 - 0.0066). **(G)** Migration directionality of MDA-MB-231 cells treated with DMSO (CTRL) or various concentrations (0.5, 1 or 2 µM) of R428 obtained from respectively 143, 127, 153 and 114 cells in 4 independent experiments (ns > 0.05, *p = 0.015).
All graphs represent mean ± s.d.

Supplementary fig.2

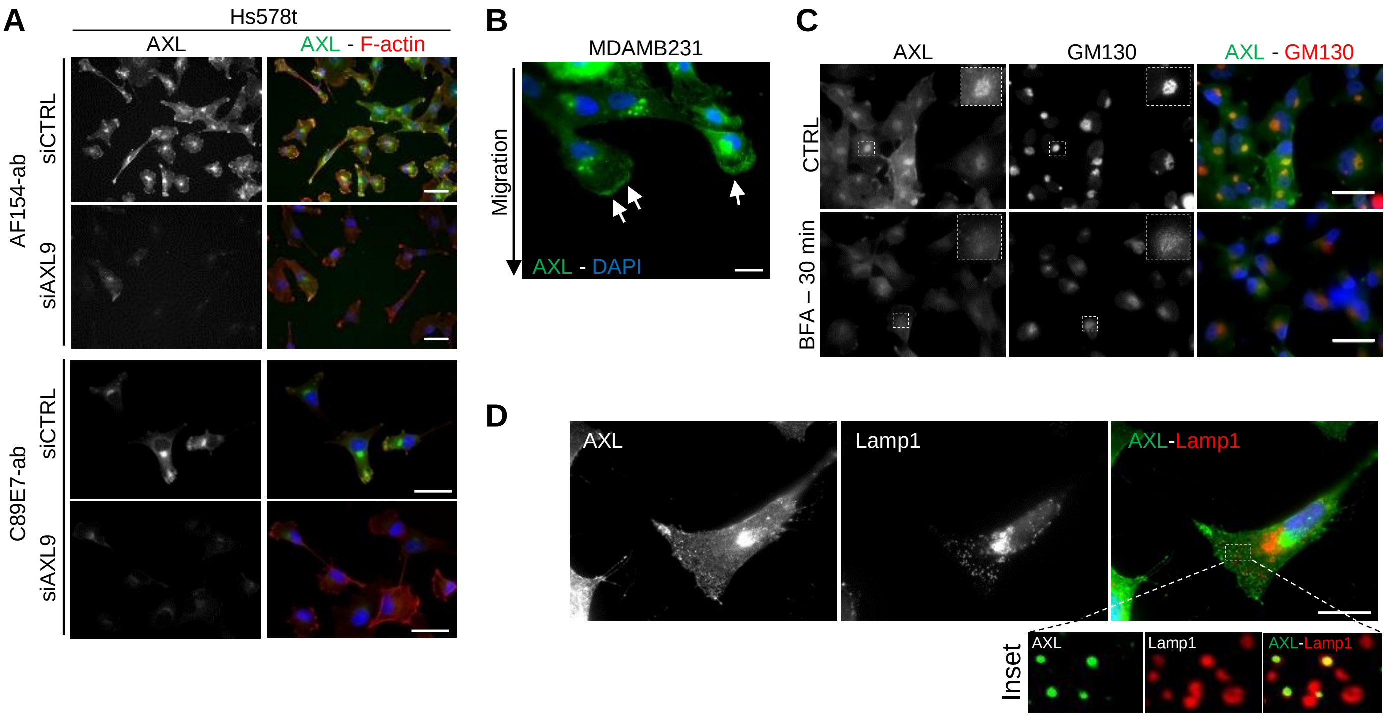

Supplementary fig.2 | (A) Hs578t cells stained for AXL (AF174 or C89E7 antibodies, green) and F-actin (red) three days after transfection with CTRL, AXL9 and AXL10 siRNA. **(B)** AXL staining on MDA-MB-231 cells six hours following wound healing. Arrows point to AXL enrichment at the front of migrating cells. **(C)** AXL (green) and GM130 (red) staining of Hs578t cells treated with DMSO or brefeldin-A (BFA) at 10 µg/ml for 30 min. **(D)** AXL (green) and Lamp1 (red) staining of Hs578t cell. Scale bars, 20 µm.

Supplementary fig.3

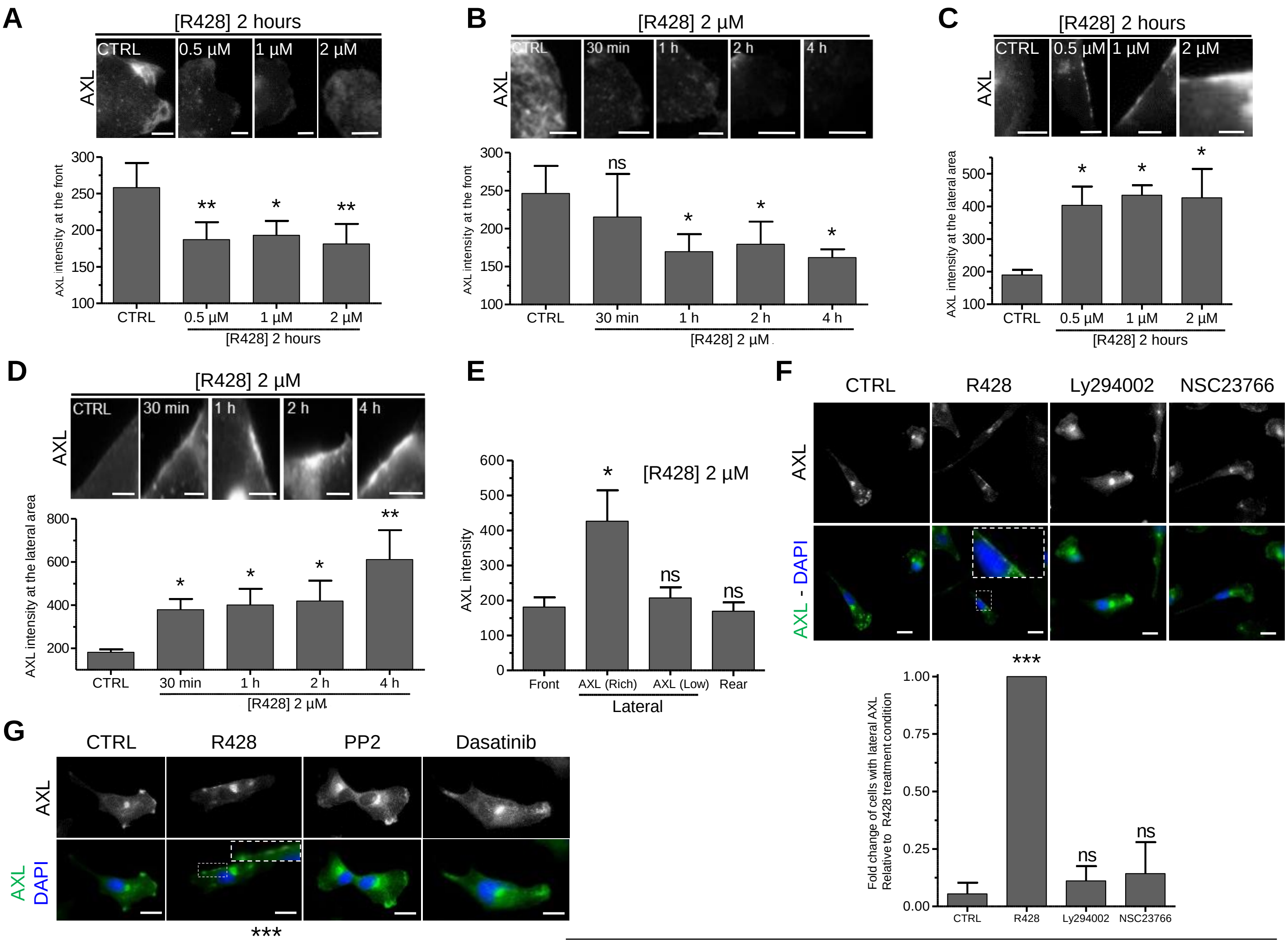

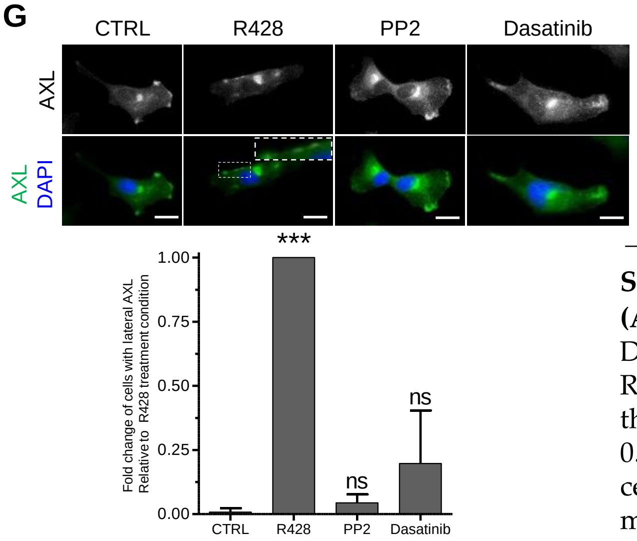

Supplementary fig.3 | **(A)** AXL intensity at the front of Hs578t cells treated with DMSO (CTRL) or various concentrations (0.5, 1 or 2 µM) of R428 during 2 hours obtained from 39, 31, 26 and 39 cells in three independent experiments, respectively (*p = 0.0362, **p = 0.0078 - 0.0032). **(B)** AXL intensity at the lateral area of Hs578t cells treated with DMSO (CTRL) or with 2 µM R428 during 30 min, 1h, 2h or 4h obtained from 38, 30, 32, 38 and 34 cells in three independent experiments, respectively (ns > 0.5, *p = 0.025 - 0.049 - 0.012). **(C)** AXL intensity at the lateral area of Hs578t cells treated with DMSO (CTRL) or various concentrations (0.5, 1 or 2 µM) of R428 during 2 hours obtained from 39, 31, 26 and 39 cells in three independent experiments, respectively (*p = 0.019 - 0.010 - 0.041). **(D)** AXL intensity at the lateral area of Hs578t cells treated with DMSO (CTRL) or with 2 µM R428 during 30 min, 1h, 2h or 4h obtained from 38, 30, 32, 38 and 34 cells in three independent experiments, respectively (*p = 0.016 - 0.043 - 0.036, **p = 0.0055). **(E)** AXL intensity at the front, lateral area and rear of Hs578t cells treated with 2 µM R428 during 2h obtained from 37 cells in three independent experiments (ns > 0.05, *p = 0.010). **(F)** AXL staining (upper panel) and AXL localization quantification (lower panel) of Hs578t cells treated with DMSO (CTRL) or with R428, Ly294002 (PI3K inhibitor) or NSC23766 (inhibitor of Rac1-GEF) obtained from 213, 226, 161 and 176 cells in three or four independent experiments, respectively (ns > 0.05, ***p < 0.001). **(G)** AXL staining (upper panel) and AXL localization quantification (lower panel) of Hs578t cells treated with DMSO (CTRL) or with R428, PP2 (SRC inhibitor) and Dasatinib (SRC inhibitor) obtained from 210, 214, 191 and 205 cells in four independent experiments, respectively (ns > 0.05, ***p < 0.001).
Scale bars, 20 µm. All graphs represent mean ± s.d.

Supplementary fig.4

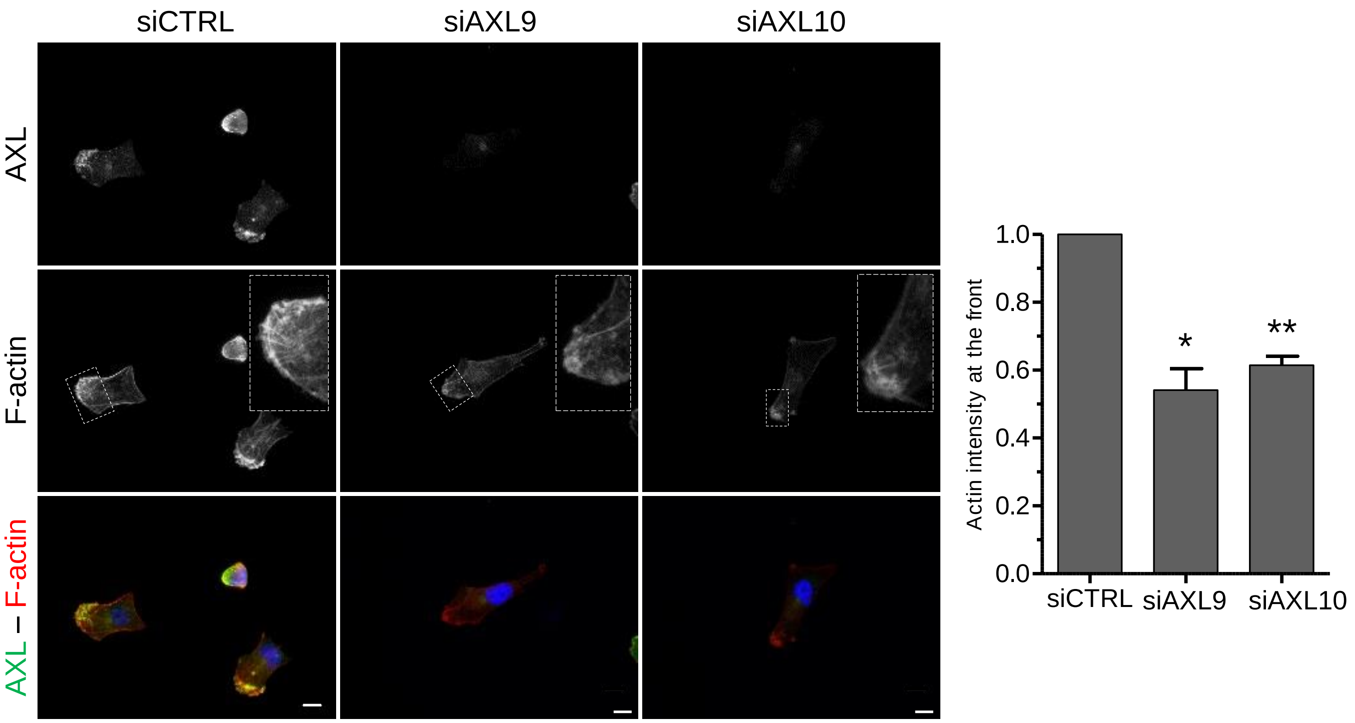

Supplementary fig.4 | AXL (green) and F-actin (red) staining (Left panel) and quantification of F-actin intensity (Right panel) at the front of Hs578t cells three days after transfection with CTRL, AXL9 and AXL10 siRNA, obtained from 47, 32 and 35 cells in three independent experiments, respectively (*p = 0.019, **p = 0.0049).
Scale bars, 20 µm. The graph represents mean ± s.d.
